# Activation of Arp2/3 Complex: Addition of the First Subunit of the New Filament by a WASP Protein Triggers Rapid ATP Hydrolysis on Arp2

**DOI:** 10.1371/journal.pbio.0020091

**Published:** 2004-04-13

**Authors:** Mark J Dayel, R. Dyche Mullins

**Affiliations:** **1**Graduate Group in Biophysics, University of CaliforniaSan Francisco, San Francisco, CaliforniaUnited States of America; **2**Department of Cellular and Molecular Pharmacology, University of CaliforniaSan Francisco, San Francisco, CaliforniaUnited States of America

## Abstract

In response to activation by WASP-family proteins, the Arp2/3 complex nucleates new actin filaments from the sides of preexisting filaments. The Arp2/3-activating (VCA) region of WASP-family proteins binds both the Arp2/3 complex and an actin monomer and the Arp2 and Arp3 subunits of the Arp2/3 complex bind ATP. We show that Arp2 hydrolyzes ATP rapidly—with no detectable lag—upon nucleation of a new actin filament. Filamentous actin and VCA together do not stimulate ATP hydrolysis on the Arp2/3 complex, nor do monomeric and filamentous actin in the absence of VCA. Actin monomers bound to the marine macrolide Latrunculin B do not polymerize, but in the presence of phalloidin-stabilized actin filaments and VCA, they stimulate rapid ATP hydrolysis on Arp2. These data suggest that ATP hydrolysis on the Arp2/3 complex is stimulated by interaction with a single actin monomer and that the interaction is coordinated by VCA. We show that capping of filament pointed ends by the Arp2/3 complex (which occurs even in the absence of VCA) also stimulates rapid ATP hydrolysis on Arp2, identifying the actin monomer that stimulates ATP hydrolysis as the first monomer at the pointed end of the daughter filament. We conclude that WASP-family VCA domains activate the Arp2/3 complex by driving its interaction with a single conventional actin monomer to form an Arp2–Arp3–actin nucleus. This actin monomer becomes the first monomer of the new daughter filament.

## Abstract 2

This paper provides the biochemical and biophysical basis for actin filament formation, necessary for cell shape and motility

## Introduction

The actin cytoskeleton determines the shape, mechanical properties, and motility of most eukaryotic cells. To change shape and to move, cells precisely control the location and timing of actin filament assembly by regulating the number of fast-growing (barbed) filament ends (Pollard et al. 2000). The actin-related protein (Arp) 2/3 complex, a seven-subunit protein complex that contains two actin-related proteins, generates these new barbed ends in response to cellular signals (Welch et al. 1998; Machesky et al. 1999; Rohatgi et al. 1999). In a process called “dendritic nucleation,” the Arp2/3 complex nucleates new actin filaments from the sides of preexisting filaments to produce a rigid and highly crosslinked filament array (Mullins et al. 1998; Machesky et al. 1999; Blanchoin et al. 2000a). Such crosslinked arrays form the core of many motile cellular structures, including the leading edges of amoeboid cells and the actin comet tails that propel endosomes and bacterial pathogens through eukaryotic cytoplasm. To understand the construction, function, and regulation of these structures, it is important to understand the molecular mechanism of Arp2/3 activation.

The Arp2/3 complex must be activated by both a member of the Wiskott–Aldrich syndrome protein (WASP) family and a preexisting actin filament before it will nucleate a new actin filament (Machesky et al. 1999; Blanchoin et al. 2001; Zalevsky et al. 2001). The structure and the orientation of the Arp2 and Arp3 subunits within the crystal structure of the complex suggest that these subunits may nucleate a new filament by forming an actin-like heterodimer that mimics the barbed end of an actin filament (Robinson et al. 2001). In the crystal structure of the unactivated complex, however, Arp2 and Arp3 are separated by 40 Å so that formation of an actin-like dimer would require a conformational change (Robinson et al. 2001). Binding of the Arp2/3 complex to both a preformed filament and a WASP-family protein is thought to drive at least part of this conformational change (Blanchoin et al. 2001; Marchand et al. 2001; Panchal et al. 2003). The Arp2/3-activating region of WASP-family proteins, also known as the VCA domain, is composed of three sequences arranged in tandem: (1) an actin-binding verprolin-homology (or V) domain (also known as a WASP-homology 2 [WH2] domain), (2) a conserved “connecting” (or C) region that interacts with both the Arp2/3 complex and monomeric actin (Marchand et al. 2001), and (3) an acidic (or A) region that binds the Arp2/3 complex. This VCA domain is both necessary and sufficient for efficient Arp2/3 activation. We and others have previously suggested that an actin monomer provided by the VCA domain to the Arp2/3 complex may drive the formation of an Arp2–Arp3–actin heterotrimer and form a nucleus for actin polymerization (Dayel et al. 2001; Marchand et al. 2001).

Both the Arp2 and Arp3 subunits of the complex bind ATP (Dayel et al. 2001). Hydrolysis of this ATP could be used to perform work, to provide a signal, or, like the guanine triphosphate (GTP) bound to the α subunit of tubulin heterodimers, may simply stabilize a protein fold. On conventional actin, ATP hydrolysis is a timing mechanism that promotes construction of dynamic and polarized filament networks. Actin rapidly hydrolyzes ATP upon polymerization (Blanchoin and Pollard 2002) and releases bound phosphate several hundred seconds later (Melki et al. 1996). ATP hydrolysis and phosphate dissociation do not cause immediate filament disassembly, but enable interaction with depolymerizing factors such as cofilin (Blanchoin and Pollard 1999). ATP hydrolysis by actin thereby determines the overall rate of filament turnover.

We show here that the Arp2/3 complex rapidly hydrolyzes ATP on the Arp2 subunit upon filament nucleation. There are several events in the Arp2/3 nucleation reaction that might trigger ATP hydrolysis on Arp2: (1) binding of VCA to the Arp2/3 complex, (2) binding of VCA-Arp2/3 to the side of a preformed filament, (3) binding of a VCA-tethered actin monomer to the Arp2/3 complex, or (4) binding of a second or third actin monomer to form a stable daughter filament. We find that ATPase activity requires the combination of a preformed actin filament, a VCA domain, and an actin monomer, but does not require actin polymerization. This indicates that hydrolysis is triggered relatively early in the nucleation reaction—before completion of a stable daughter filament. Capping the pointed ends of actin filaments also stimulates Arp2 to rapidly hydrolyze ATP in the absence of monomeric actin and VCA and without branch formation. Thus, ATP hydrolysis on Arp2 is stimulated directly by interaction with conventional actin, presented to the complex either as a monomer attached to the VC domain of the WASP-family protein or as one of the subunits making up the pointed end of a preformed filament. To our knowledge this is the first direct evidence that the monomer supplied by the VCA domain is the first monomer of the new daughter filament. From these observations we propose a model for the mechanism of Arp2/3 complex activation by WASP-family proteins.

## Results

### γ-^32^P-AzidoATP Can Be Covalently Crosslinked to Arp2 and Arp3 with Approximately Equal Efficiency

**Figure 1:**
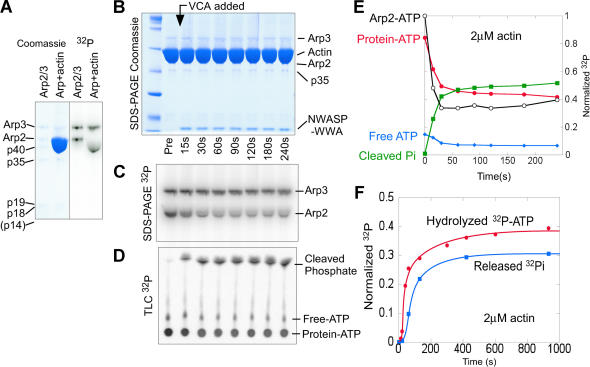
Arp2 Hydrolyzes ATP Rapidly upon Filament Nucleation (A) Arp2/3 (2 μM) was covalently crosslinked to γ-^32^P-AzidoATP by exposure to UV light. Both Arp2 and Arp3 crosslink with approximately equal efficiency (lane 1). Addition of 100-fold excess monomeric actin (lane 2) distorts the shape of the Arp2 band, but the Arp2 signal remains separate and quantifiable. (B–E) γ-^32^P-AzidoATP-Arp2/3 (20 nM) was mixed with 2 μM monomeric actin in polymerization buffer. Samples were taken before and at indicated times after the addition of 750 nM VCA, which initiates rapid actin-filament nucleation by the Arp2/3 complex. (B) Subunits were separated by SDS-PAGE and stained with Coomassie. (C) ^32^P signal shows remaining uncleaved γ-^32^P on Arp2 and Arp3 subunits. Arp2 rapidly loses γ-^32^P after addition of VCA. (D) Cleaved γ-^32^P was separated from free ^32^P-ATP and protein-^32^P-ATP by TLC. (E) Quantitation of (B) to (D): Protein-ATP (closed circle), Cleaved Pi (closed square), Free ATP (closed diamonds), and Arp2-ATP from SDS-PAGE (open circle, normalized separately). (F) Arp2 releases phosphate soon after ATP hydrolysis. Reaction conditions were the same as (B)–(E), but with the addition of 2 mM maltose and 2 U/ml maltose phosphorylase. Timepoints were quenched into formic acid and assayed by TLC. Hydrolyzed ^32^P-ATP was quantified from the decrease in protein conjugated ^32^P, and released ^32^P was quantified from the ^32^P-glucose phosphate produced.

Previously we used sodium dodecyl sulphate polyacrylamide gel electrophoresis (SDS-PAGE) to show that UV irradiation covalently crosslinks α-^32^P-8-AzidoATP to the Arp2 and Arp3 subunits of the Arp2/3 complex (Dayel et al. 2001). Here we crosslink γ-^32^P-AzidoATP instead of α-^32^P to Arp2 to measure ATPase activity. Using SDS-PAGE, we can separate the subunits and simultaneously monitor cleavage of the labeled γ-phosphate from ATP bound to both Arp2 and Arp3. This technique allows us to measure ATP hydrolysis specifically on the Arp2/3 complex in spite of a 100-fold molar excess of actin, which also binds and hydrolyzes ATP. We crosslinked γ-^32^P-AzidoATP to the Arp2/3 complex by brief (9 s) exposure to UV light. In the presence of γ-^32^P-AzidoATP at concentrations above the K_D_ for ATP (Dayel et al. 2001), γ-^32^P-AzidoATP crosslinks to both Arp2 and Arp3 with approximately equal efficiency (Figure 1A). Addition of large amounts of monomeric actin to the labeled Arp2/3 distorts the shape of the Arp2 band, but the ^32^P signal from Arp2 remains separately quantifiable, and the magnitude is unaffected (Figure 1A). The efficiency of crosslinking for both Arp2 and Arp3 is approximately 10% (unpublished data); therefore, only 1% of the Arp2/3 complex has γ-^32^P-AzidoATP crosslinked to both Arp2 and Arp3. For simplicity, we refer to this partially crosslinked Arp2/3 complex as γ-^32^P-AzidoATP-Arp2/3. Reactions using γ-^32^P-AzidoATP-Arp2/3 are performed in the presence of 100 μM ATP, to occupy the noncrosslinked sites and ensure 100% of the Arp2/3 complex is active.

### Arp2 Hydrolyzes ATP Rapidly upon Actin Filament Nucleation

**Figure 3:**
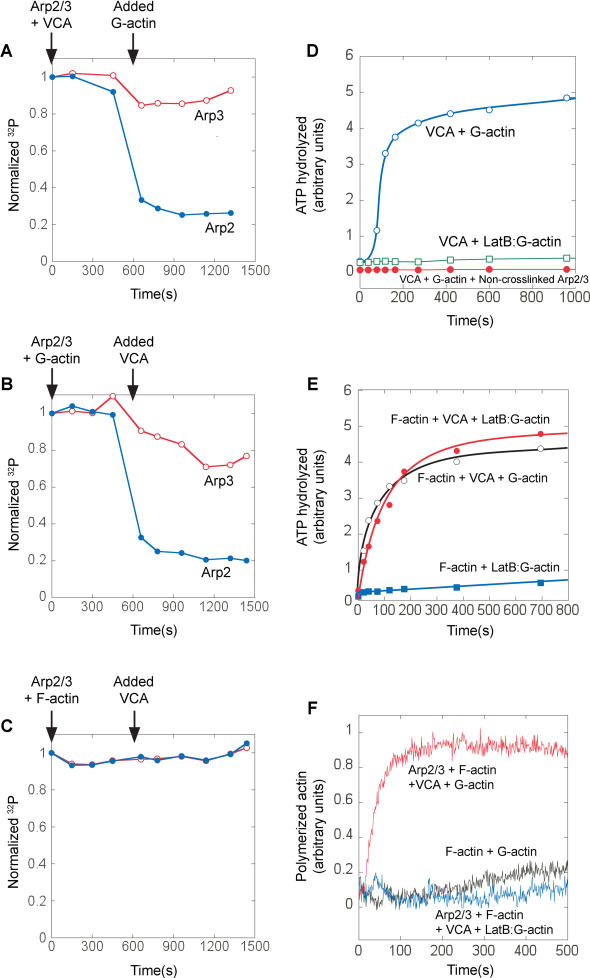
A Single Actin Monomer, in the Presence of Actin Filaments and VCA, Stimulates ATP Hydrolysis on Arp2, without Requiring Actin Polymerization (A–C) Remaining unhydrolyzed γ-^32^P-AzidoATP on Arp2 (closed circle) and Arp3 (open circle) was quantified to assay ATP hydrolysis (same conditions as Figure 1B–1D). γ-^32^P-AzidoATP-labeled Arp2/3 (20 nM) was mixed at indicated times with either 750 nM VCA then 2 μM G-actin (A), 2 μM G-actin then 750 nM VCA (B), or 2 μM F-actin then 750 nM VCA (C). (D) Latrunculin B (open square) inhibits the ability of VCA plus monomeric actin (open circle) to stimulate ATP hydrolysis on the Arp2/3 complex in the absence of actin filaments. Also, ^32^P ATP hydrolysis signal requires covalent crosslinking to Arp2/3. Arp2/3 was mixed with 6 μM γ-^32^P-AzidoATP and exposed to UV either before (closed circle) or after (open circle) the addition of excess (2 mM) unlabeled ATP. Excess ATP added before the UV exposure prevents crosslinking and abolishes the ATP hydrolysis signal, indicating that all the ^32^P ATP hydrolysis signals measured are due to ATP hydrolysis on Arp2/3 and not from ATP hydrolysis on actin. (E and F) In the presence of phalloidin-stabilized actin filaments, actin monomers are prevented from polymerizing by Latrunculin B, but still stimulate ATP hydrolysis on the Arp2/3 complex. 20 nM γ-^32^P-AzidoATP–labeled Arp2/3 was premixed with 1 μM phalloidin-stabilized actin filaments. The reaction was initiated by mixing with 750 nM N-WASP VCA, 1 μM G-actin and 4 μM Latrunculin B as indicated, cleaved γ-^32^P was assayed by phosphomolybdate extraction (E), and separately, actin polymerization was monitored by pyrene–actin fluorescence (F).

**Figure 4:**
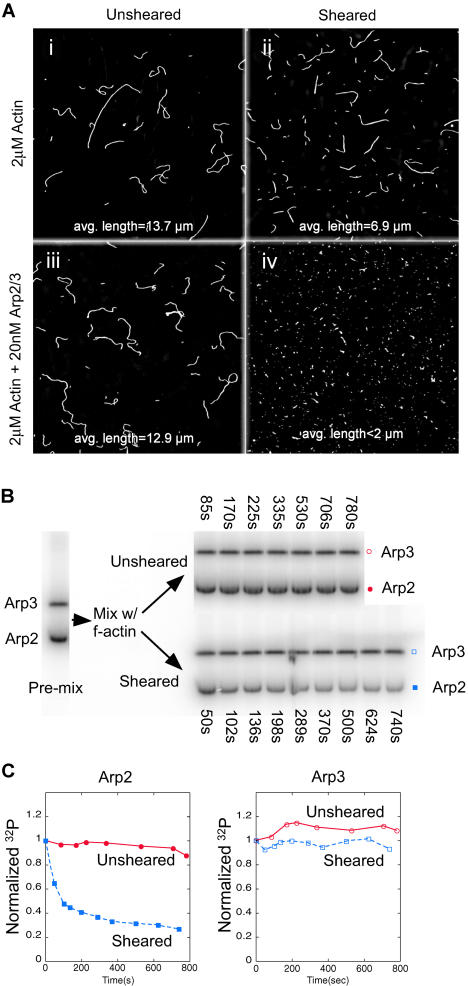
Pointed-End Filament Capping Is Sufficient to Stimulate ATP Hydrolysis on Arp2 in the Absence of VCA (A) The Arp2/3 complex prevents actin filament reannealing by capping the pointed ends. The length distribution of 2 μM Alexa-488 phalloidin-stabilized actin filaments is unaffected in the absence (i) or presence (iii) of 20 nM Arp2/3 complex. (ii) 5 min after shearing the filaments, filaments have begun to reanneal in the absence of the Arp2/3 complex, but 20 nM Arp2/3 complex (iv) maintains short filaments, preventing reannealing by capping filament pointed ends. (B) ATP hydrolysis on Arp2 is stimulated by pointed-end capping. Crosslinked γ-^32^P-AzidoATP-Arp2/3 (20 nM) was mixed with 2 μM phalloidin-stabilized actin filaments. The mixture was split in two and one sample was sheared. Timepoints were taken as shown. (C) Uncleaved ^32^P on Arp2 (unsheared [closed circle] and sheared [closed square]) and Arp3 (unsheared [open circle] and sheared [open square]) were quantified from (B). Arp2 rapidly hydrolyzes bound ATP upon filament pointed-end capping.

We mixed 20 nM γ-^32^P-AzidoATP-Arp2/3 with 2 μM monomeric actin in polymerization buffer and initiated polymerization by adding 750 nM VCA, which activates rapid actin filament nucleation by the Arp2/3 complex (t_½ actin polymerization_ ≈ 20 s; unpublished observations). (Unless otherwise stated, VCA refers to 6-histidine [6His]-N-WASP-VCA [398-502]. Cleavage of the 6His tag did not affect the kinetics of Arp2/3-mediated actin polymerization [unpublished data].) We assayed timepoints both by SDS-PAGE and thin-layer chromatagraphy (TLC) during the same reaction to monitor remaining and cleaved ^32^P, respectively (Figure 1B–1D; quantified in Figure 1E). ATP is hydrolyzed by the Arp2/3 complex at the earliest timepoints after the addition of VCA (monitored by ^32^P cleavage) and cleavage has ceased by 90 s (Figure 1D). SDS-PAGE analysis separates the subunits and shows that the γ-^32^P is cleaved rapidly from Arp2 upon addition of VCA, but not significantly from Arp3 (Figure 1C). The kinetics of ATP hydrolysis assayed by SDS-PAGE match the kinetics of phosphate cleavage by TLC (Figure 1E). Since the nucleation reaction is autocatalytic, the rate increases over time, and therefore it is not possible to derive an exact ATPase rate constant from our data. However, we can define a conservative lower bound: k_hyd_ > 0.1 s^–1^, noting that the true rate constant is probably much higher. Isolated Arp2/3 complex in polymerization buffer shows very slow spontaneous cleavage of γ-^32^P from both Arp2 and Arp3 (<1 × 10^–4^ s^–1^) (unpublished data). As a control, ^32^P-ATP hydrolysis is only seen when the Azido-ATP is covalently crosslinked to the Arp2/3 complex (Figure 3D, compare open and closed circles) indicating that the signal is due only to hydrolysis of ATP covalently bound to the Arp2/3 complex and not due to ATP hydrolysis by polymerizing actin. This is further confirmed by observations of ATP hydrolysis on the Arp2/3 complex under conditions where no actin polymerization takes place (Figure 3E and 3F; Figure 4).

### Phosphate Release by Arp2 Lags Hydrolysis by Approximately 40 s

To investigate the kinetics of phosphate release fromArp2/3 during the polymerization reaction, we added maltose and maltose phosphorylase to the reaction. In the presence of ^32^P-labeled Arp2/3 complex, maltose phosphorylase conjugates the ^32^P-orthophosphate released from Arp2 to a hydrolyzed maltose molecule to make ^32^P-glucose phosphate. The phosphate from adenosine diphosphate-inorganic phosphate (ADP-Pi)-bound Arp2 is inaccessible to the enzyme and remains unconjugated orthophosphate. We quantified hydrolyzed ^32^P-ATP and released phosphate by TLC (Figure 1F). Phosphate release from Arp2 lags behind ATP hydrolysis by approximately 40 s.

### The Rate of Filament Nucleation Matches the Rate of ATP Hydrolysis by Arp2

**Figure 2:**
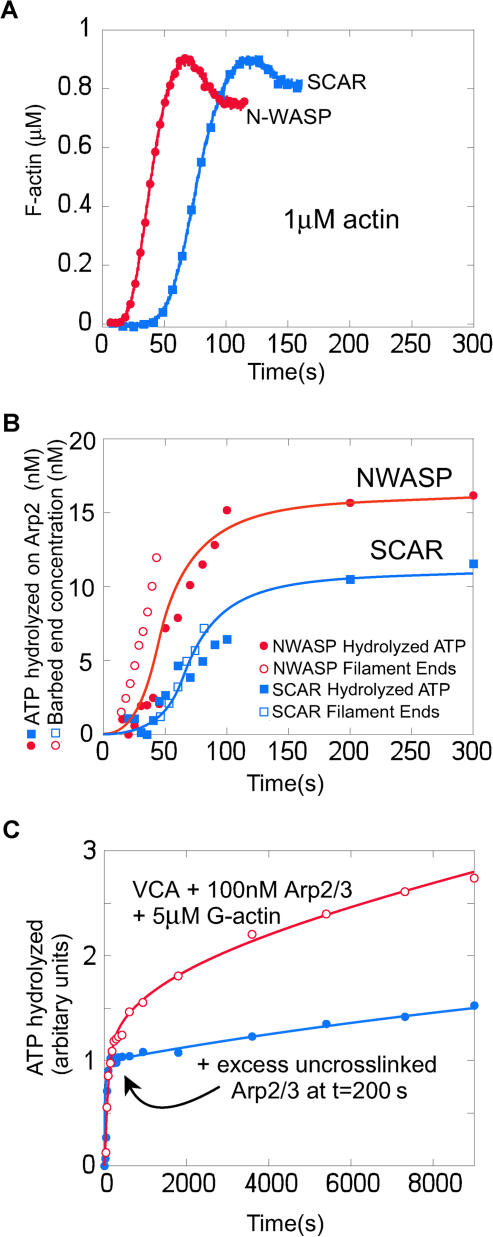
ATP Hydrolysis by Arp2 Coincides with Nucleation of New Actin Filaments, and Not Filament Debranching (A, B) The kinetics of nucleation were slowed by using only 1 μM monomeric actin (compared to 2 μM for Figure 1). γ-^32^P-AzidoATP-Arp2/3 (20 nM) was mixed with either 750 nM N-WASP WWA (closed circle) or Scar1 WA (closed square) and 1 μM 7% pyrene-labeled monomeric actin. (A) Actin polymerization measured by pyrene fluorescence. (B) The concentration of new filament ends (open symbols) was calculated from the polymerization data in a model-independent way (see Methods and Materials), and Arp2-ATP hydrolysis (closed symbols) was measured under the same reaction conditions for both N-WASP WWA (open and closed circles) and Scar1 WA (open and closed squares). (C) ATP hydrolysis on Arp2 does not accompany filament debranching. Using a large excess (100 nM) of γ-^32^P-AzidoATP-Arp2/3 creates a slow hydrolysis phase that follows the rapid nucleation phase. The slow phase of ATP hydrolysis can be inhibited by excess (1.5 μM) uncrosslinked Arp2/3 added at t = 200 s, showing that the slow phase of ATP hydrolysis is from Arp2/3 being recruited from solution and not from that already incorporated in branches.

To determine whether ATP hydrolysis on Arp2 is coupled to filament nucleation, we varied the rate of nucleation and looked to see whether the rate of ATP hydrolysis by Arp2 varied accordingly. We varied the nucleation rate by using N-WASP and Scar1 VCA domains, which stimulate different rates of Arp2/3 complex-dependent actin nucleation (Zalevsky et al. 2001). To slow the nucleation reaction and allow more accurate kinetic measurements, we used only 1 μM monomeric actin. We used pyrene–actin polymerization data (Figure 2A) to calculate the concentration of barbed ends produced over time (Figure 2B, open symbols) (see Methods and Materials; Zalevsky et al. 2001). Note that this calculation is model-independent and simply uses the established kinetic parameters for actin polymerization and the change in the amount of monomeric and filamentous actin over time measured from the pyrene–actin curves. The same reagents were used to monitor ATP hydrolysis by Arp2 under the same conditions. We used loss of γ-^32^P labeling as a probe for ATP hydrolysis and scaled the initial labeling intensity to the Arp2/3 concentration used in the reaction (20 nM) to calibrate the stoichiometry of ATP hydrolyzed by Arp2 (Figure 2B). Using Scar1 VCA instead of N-WASP VCA halves both the rate of nucleation of actin filaments and the rate of ATP hydrolysis on Arp2.

We note that the total amount of Arp2 that hydrolyzes ATP in the polymerization reaction is 30% less for Scar1 VCA than for N-WASP VCA, which we interpret as 30% fewer filaments produced. Although it is possible to calculate the *rate* of end production from the pyrene–actin polymerization curve in a model-independent way, it is not possible to calculate the *total* number of barbed ends produced, since once polymerization reaches equilibrium, the pyrene–actin curve will not change even if new barbed ends continue to be produced. From the ATP hydrolysis data, therefore, the Arp2/3 complex produces filament ends more slowly when activated by Scar1, and under our conditions, the reaction ends when monomeric actin is depleted by incorporation into the new filaments. Fewer total filaments are therefore produced by the less active VCA domain.

### ATP Hydrolysis on Arp2 Does Not Accompany Filament Debranching

A previous study claimed that ATP hydrolysis on Arp2 occurs very slowly (t_1/2_ ≈ 800 s), coincident with filament debranching (Le Clainche et al. 2003). Le Clainche et al. (2003) used a much higher concentration of Arp2/3 complex (100 nM) in their assays than the 5 nM Arp2/3 complex that they estimate was used up during their polymerization reaction. Using these conditions, we find that Arp2/3 complex hydrolyses ATP in two discrete phases: a fast (nucleation) phase, followed by a slow, approximately linear phase (Figure 2C, open symbols). This slow phase does not plateau within 6000 s and is similar to the data presented in Le Clainche et al. (2003). To demonstrate that this slow ATP hydrolysis is not due to the Arp2/3 complex hydrolyzing ATP upon debranching, we added an excess of unlabeled Arp2/3 complex into solution at t = 200 s, after the polymerization phase is complete. This unlabeled Arp2/3 complex competes for nucleating factors with γ-^32^P-AzidoATP-Arp2/3 in solution, but it does not compete with γ-^32^P-AzidoATP-Arp2/3 already incorporated in branches. Addition of excess unlabeled Arp2/3 complex at t = 200 s inhibits the slow phase of ATP hydrolysis (Figure 2C, closed symbols), indicating that the slow phase is due to ATP hydrolysis on Arp2/3 complex being recruited from solution and not due to ATP hydrolysis on Arp2/3 complex already in branches. This slow ATP hydrolysis probably represents a low rate of filament nucleation by the excess unused Arp2/3 complex, the rate of nucleation being limited by the low monomeric actin concentration that remains after most of the actin has polymerized.

### Both VCA and Monomeric Actin Are Required to Stimulate ATP Hydrolysis by Arp2 during the Polymerization Reaction

Although the kinetics of ATP hydrolysis on Arp2 match the kinetics of actin polymerization, these data do not rule out the possibilities that VCA alone or the filamentous actin created during the polymerization reaction stimulates the ATPase activity independent of nucleation. To more specifically determine what stimulates ATP hydrolysis on Arp2, we varied the order of addition of the components that initiate the polymerization reaction. Incubation of the Arp2/3 complex with VCA does not induce ATP hydrolysis by the complex until monomeric actin is added to the reaction (Figure 3A), showing that VCA alone does not stimulate the ATPase activity. Similarly, monomeric actin alone does not stimulate the Arp2/3 complex to hydrolyze ATP until the addition of VCA (Figure 3B). To test whether actin filaments themselves stimulate Arp2/3 ATP hydrolysis, we used phalloidin-stabilized actin filaments to ensure that no monomeric actin would be present and took care not to shear the filaments in order to reduce the number of free pointed ends. ATP hydrolysis is not stimulated on the Arp2/3 complex by filamentous actin, even in presence of VCA (Figure 3C). As controls, we found that neither 5 μM phalloidin nor 20 mM phosphate inhibit the kinetics of ATP hydrolysis by Arp2 during the polymerization reaction (unpublished data).

When Arp2/3 concentration is low (20 nM), and nucleation is rapid (using N-WASP VCA), initiation of the polymerization reaction causes striking and near-complete ATP hydrolysis on Arp2 (approximately 80%, i.e., approximately 16 nM; Figure 3B and 3C). We detect a small amount of ATP hydrolysis on Arp3 with similar kinetics but much lower stoichiometry (10%–20%). The decrease is not caused by the dilution effect of adding the second component (approximately 4%), which is already compensated for in the data presented.

### In the Presence of Both VCA and Actin Filaments, a Nonpolymerizable Actin Monomer Is Sufficient to Trigger Rapid ATP Hydrolysis on Arp2

The timing and stoichiometry of ATP hydrolysis and the combination of factors required to stimulate it suggest that Arp2 hydrolyzes ATP during the filament nucleation reaction. Kinetic and light-microscopy data indicate that most or all Arp2/3-dependent filament nucleation occurs from Arp2/3 complex bound to the sides of filaments produced earlier in the polymerization reaction (Blanchoin et al. 2000a, 2001; Zalevsky et al. 2001). To test whether filament side-binding is necessary for ATP hydrolysis on Arp2, we blocked filament formation with the actin-monomer binding toxin, Latrunculin B. Latrunculin B binds to monomeric actin and prevents it polymerizing, but does not affect its binding to VCA (R. D. Mullins and A. E. Kelly, unpublished data). The combination of VCA and Latrunculin B–actin monomers does not stimulate ATP hydrolysis on Arp2/3 complex (Figure 3D, open squares), nor do preformed, phalloidin-stabilized actin filaments and Latrunculin B–actin monomers without VCA (Figure 3E, filled squares). In the presence of preformed actin filaments and VCA, however, Latrunculin B–actin monomers stimulate rapid ATP hydrolysis on Arp2/3 (Figure 3E, filled circles) without actin polymerization (Figure 3F). Table 1 summarizes the requirements for stimulation of ATP hydrolysis on Arp2. These data indicate that during the nucleation reaction, actin filament side-binding by Arp2/3 complex is a prerequisite for VCA and monomeric actin to stimulate ATP hydrolysis on Arp2. The observation that polymerization of the daughter filament is unnecessary implies that the VCA-mediated interaction of a single actin monomer with the Arp2/3 complex is the trigger for ATP hydrolysis on Arp2.

**Table 1:**
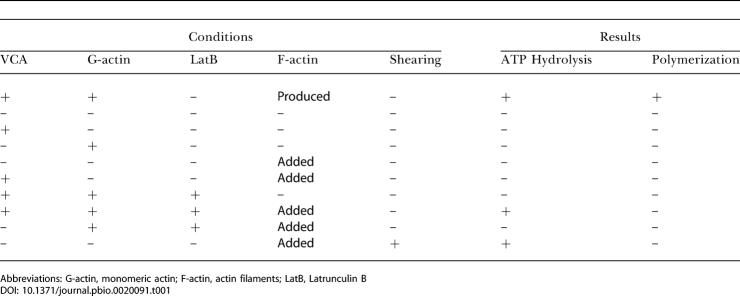
Requirements to Stimulate ATP Hydrolysis on the Arp2 Subunit of Arp2/3 Complex

Abbreviations: G-actin, monomeric actin; F-actin, actin filaments; LatB, Latrunculin B

### Pointed-End Capping by the Arp2/3 Complex Stimulates Rapid ATP Hydrolysis by Arp2 in the Absence of Either Branch Formation or a WASP-Family VCA Domain

The Arp2/3 complex is known to cap the pointed ends of preformed actin filaments in vitro, inhibiting both polymerization and depolymerization from the pointed ends of gelsolin-capped filaments (Mullins et al. 1998). The Arp2/3 complex does not cap the barbed ends of actin filaments and does not affect the rate of addition of monomers from the barbed ends of spectrin-capped filaments (unpublished data). We speculated that the way the Arp2/3 complex caps a free-filament pointed end in solution might mimic the way the Arp2/3 complex anchors the pointed end of the new daughter filament in a branch. If the actin monomer that triggers ATP hydrolysis during nucleation is the first monomer of the daughter filament, pointed-end capping, like nucleation, should drive interaction with this monomer and trigger ATP hydrolysis on Arp2. To test this, we sheared preformed, phalloidin-stabilized actin filaments in the presence of the Arp2/3 complex. Mechanical shearing fragments long actin filaments into many short filaments, creating many new filament ends that rapidly reanneal to produce long filaments again (Murphy et al. 1988). This reannealing process is blocked by proteins that cap filament ends (Andrianantoandro et al. 2001). Without shearing, the addition of 20 nM Arp2/3 complex does not alter the length distribution of phalloidin-stabilized actin filaments (Figure 4A, compare [i] and [iii]). After shearing in the presence of 20 nM Arp2/3 complex, pointed-end capping by the Arp2/3 complex blocks reannealing and results in significantly shorter filaments (Figure 4A, compare [ii] and [iv]). No branches form within this time—it takes several hours for even a few branches to assemble under these conditions (unpublished data). To assay for ATP hydrolysis by the complex, we incubated γ-^32^P-AzidoATP-Arp2/3 complex with actin filaments under the same conditions as the microscopy experiment. We split the mixture into two parts, sheared one half, and took timepoints to assay for ATP hydrolysis from both samples (Figure 4B; quantified in Figure 4C). No ATP hydrolysis occurs in the unsheared condition, confirming that binding to the sides of actin filaments is not sufficient to stimulate ATP hydrolysis. ATP hydrolysis occurs rapidly in the sheared condition and occurs only on Arp2 (Figure 4C). Since this occurs well before any branches form, pointed-end capping by the Arp2/3 complex is sufficient to stimulate ATP hydrolysis on Arp2 not only in the absence VCA, but also in the absence of filament side-binding.

## Discussion

Conventional actin and all actin-related proteins share a conserved nucleotide binding pocket. Actin monomers bind ATP but do not hydrolyze it until they are induced to polymerize. Actin polymerization triggers rapid ATP hydrolysis, followed by a slow release of cleaved phosphate from the filament (Blanchoin and Pollard 2002). Arp2 also hydrolyzes its bound ATP, and we find that the conditions that promote ATP hydrolysis and the kinetics of the reaction are remarkably similar to those of conventional actin. In the presence of VCA and actin filaments, monomeric actin stimulates ATP hydrolysis on Arp2 (Table 1). We also find that binding of the Arp2/3 complex to the pointed end of a preformed actin filament is sufficient to trigger Arp2 ATP hydrolysis, even in the absence of VCA. The stimulation of Arp2 ATPase activity by both filament pointed ends and by actin monomers under nucleating conditions suggests that the geometry of the Arp2/3–actin interaction is the same in both cases.

Interaction between the Arp2/3 complex and conventional actin can occur in three distinct ways: (1) the Arp2/3 complex binds the sides of preformed actin filaments; (2) the Arp2/3 complex binds to the pointed ends of filaments, either by remaining associated with the daughter filament following nucleation or by capping preformed pointed ends; and (3) the Arp2/3 complex may interact with an actin monomer bound to the VCA domain of a WASP-family protein. There is abundant experimental evidence for filament side- and pointed-end binding by the complex (Mullins et al. 1998; Blanchoin et al. 2000a, 2001; Amann and Pollard 2001a, 2001b). Evidence that a VCA-bound actin monomer interacts with the Arp2/3 complex is more circumstantial and is supported by four observations: (1) VCA domains can simultaneously bind both the Arp2/3 complex and monomeric actin (Marchand et al. 2001; Panchal et al. 2003); (2) removal of the actin monomer-binding WH2 (V) domain from a WASP-family protein severely decreases the efficiency of Arp2/3 activation (Marchand et al. 2001); (3) kinetic modeling suggests that the Arp2/3 complex requires monomeric actin to form a filament nucleus (Zalevsky et al. 2001); and (4) Arp2/3-dependent nucleation is not limited to the end of the mother filament (Amann and Pollard 2001a), indicating that the VCA-bound actin monomer does not incorporate into the mother filament. Two of the three interactions between the Arp2/3 complex and conventional actin—nucleation and pointed-end capping—are thought to be mediated by the actin-related subunits, analogous to actin–actin interactions in a filament. Both interactions stimulate rapid ATP hydrolysis by Arp2.

**Figure 5:**
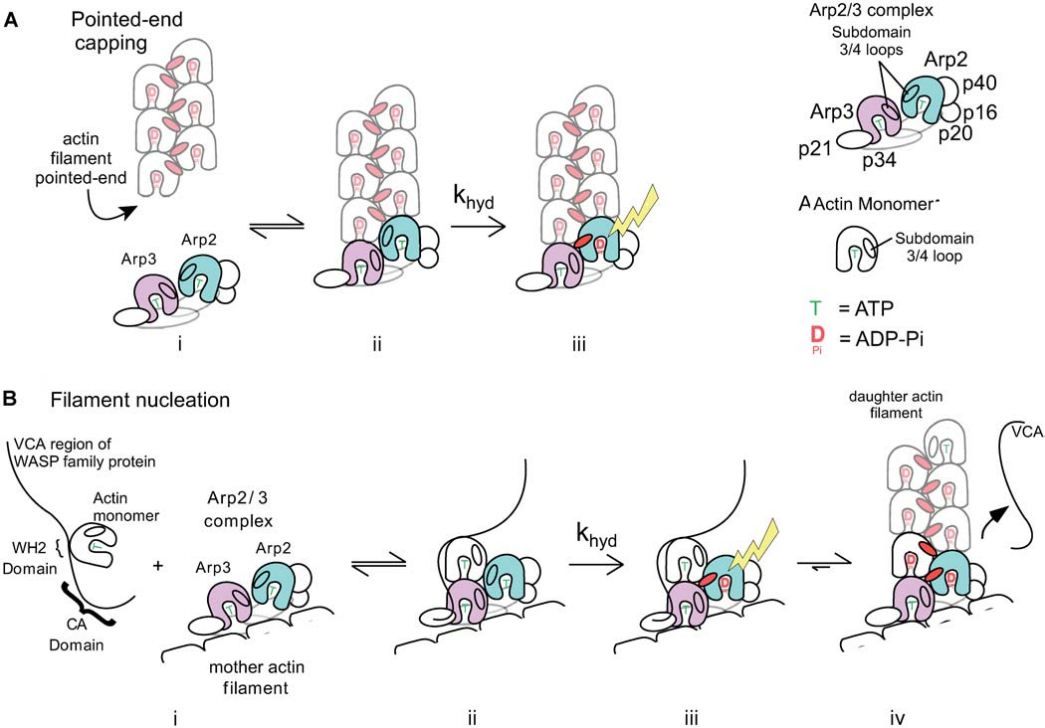
Model for Activation of ATP Hydrolysis on the Arp2/3 Complex and Mechanism by which WASP-Family Proteins Activate the Arp2/3 Complex to Nucleate New Actin Filaments (A) Filament pointed-end capping stimulates ATP hydrolysis on Arp2 without branch formation. (i) Arp2 and Arp3 are separated when the Arp2/3 complex is free in solution. (ii) Upon pointed-end capping, the binding energy of the actin-Arp2/3 interface drives Arp2 and Arp3 together and (iii) a conformational change on Arp2 (shown by the red the subdomain 3/4 loop flipping out) triggers ATP hydrolysis by Arp2 (filament pointed-end capping is probably not a significant function of the Arp2/3 complex in vivo). (b) A VCA-bound actin monomer drives the activation of the Arp2/3 complex and stimulates ATP hydrolysis on Arp2. (i) The Arp2/3 complex must first be bound to the side of an actin filament, and an actin monomer is bound to the VC domain of the WASP-family protein. (ii) The VC domain of the WASP-family protein docks the first monomer of the daughter filament onto the Arp2/3 complex, stabilizing the Arp2–Arp3–actin interaction and promoting the active conformation of the complex. (cf. Aii). (iii) The active conformation of the Arp2–Arp3–actin monomer triggers a conformational change on Arp2 and ATP hydrolysis by the subunit. (iv) Actin polymerizes from the activated Arp2/3 complex. ATP hydrolysis by Arp2 may promote dissociation of the CA domain of the WASP-family protein from the Arp2/3 complex, aided by actin polymerization, which competes its WH2 domain from the first actin monomer.

Based on sequence conservation and biochemical similarities, ATP hydrolysis on Arp2 is probably driven by a mechanism similar to that which stimulates ATP hydrolysis on actin. The molecular details of how polymerization activates ATP hydrolysis on conventional actin, however, are not well understood. A leading hypothesis is that a “hydrophobic plug”—a loop between subdomains 3 and 4 of actin (residues 262–274 in yeast; Kuang and Rubenstein 1997)—undocks from the monomer surface and binds to a hydrophobic cleft formed by adjacent monomers in the opposite strand of the two-start filament helix (Lorenz et al. 1993; Kuang and Rubenstein 1997). Our data are consistent with stimulation of ATP hydrolysis by docking of a hydrophobic plug sequence on Arp2 into a hydrophobic cleft created by Arp3 and the first actin monomer of the daughter filament (Figure 5). In the crystal structure of the inactive Arp2/3 complex, Arp2 and Arp3 are oriented like a pair of actin monomers in opposite strands of the two-start filament helix (Robinson et al. 2001), but they are separated by a 40 Å cleft. Our data support a model in which activation of the complex involves closure of the cleft, allowing actin to polymerize from an Arp2–Arp3 heterodimer (Kelleher et al. 1995; Robinson et al. 2001), which then remains attached to the pointed end of the new daughter filament, anchoring it to the branch (Figure 5B [iv]). Based on the geometry of the subunits in the crystal structure and the hydrophobic plug model, we expect that the Arp3–actin contact creates a pocket to bind the hydrophobic plug of Arp2 (residues 265–277 in yeast Arp2). The geometry of the interaction would stimulate the ATPase activity of Arp2, but not Arp3 (Figure 5A).

Monomeric actin does not interact directly with the Arp2/3 complex in the absence of VCA, but under conditions that promote nucleation, a single actin monomer triggers VCA-dependent ATP hydrolysis on Arp2. By analogy with capping-induced ATP hydrolysis, the monomer that triggers ATPase activity is therefore the first monomer of the new daughter filament (Figure 5B [i]–[iii]). The hydrophobic pocket formed between Arp2, Arp3, and the actin monomer would therefore promote a similar conformational change in Arp2 and stimulate ATP hydrolysis (Figure 5B [iv]).

Interaction of the Arp2/3 complex with the sides of filaments is not sufficient to trigger Arp2 ATPase activity, even in the presence of VCA. Binding of Arp2/3 to the sides of filaments is, however, required for ATP hydrolysis on Arp2 stimulated by VCA and monomeric actin. These data suggest that binding the side of an actin filament induces a conformational change in the Arp2/3 complex that enables it to interact with the actin monomer bound to VCA. The filament side-binding activity of Arp2/3 does not require the presence of the Arp2 or Arp3 subunits and can be reconstituted by a combination of the Arc2 (p34) and Arc4 (p20) subunits (Gournier et al. 2001). The Arc2 and Arc4 subunits contact both Arp2 and Arp3, and therefore filament side-binding might favor association of Arp2 and Arp3. The fact that Arp2-ATP hydrolysis induced by VCA and an actin monomer requires filament side-binding strongly suggests that all Arp2/3-generated actin filaments are born on the side of preformed filaments.

Our results disagree with a recent paper that claims that ATP hydrolysis on Arp2 is slow and accompanies filament debranching (Le Clainche et al. 2003). Using experimental conditions similar to the previous study, we observe similar slow ATP hydrolysis kinetics (Figure 2C) and show that this ATP hydrolysis occurs on Arp2/3 complex recruited slowly from solution. The slow hydrolysis does not reflect delayed ATP hydrolysis on Arp2/3 complex that had been rapidly incorporated into branches early in the experiment. ATP hydrolysis on Arp2, therefore, cannot be associated with debranching. Le Clainche et al. (2003) claim that ATP hydrolysis does not occur during nucleation and present data with a lag of several hundred seconds between computer-simulated nucleation kinetics and measured ATP hydrolysis kinetics (Figure 1B in Le Clainche et al. 2003). In this experiment, Le Clainche et al. (2003) initiate polymerization in the absence of free ATP. These conditions would deactivate up to 97% of the Arp2/3 complex (the fraction that is not crosslinked to ATP on both subunits). In our experience, removal of free ATP introduces an artificial lag in polymerization that lasts until tightly bound ATP is released from monomeric actin (1/k_ATP release_ = 330 s; Selden et al. 1999) and is free to interact with the Arp2/3 complex (unpublished data). The claim by Le Clainche et al. (2003) that the absence of free ATP does not affect ATP hydrolysis kinetics is contradicted by their observation that the ^32^P signal is unchanged by the addition of free ATP. The ^32^P signal is only equivalent to hydrolyzed ATP in the absence of free ATP. The addition of free ATP should cause the excess of uncrosslinked Arp2/3 complex to compete with the small fraction of crosslinked ^32^P-ATP-Arp2/3 complex and thereby significantly reduce the ^32^P signal. The observation that the ^32^P signal is not reduced, rather than confirming that removal of free ATP has no effect, instead confirms that contaminating ATP is present for the latter part of the “ATP-free” condition, presumably released slowly from monomeric actin. The lag in the polymerization created by the initial absence of ATP would be present in the experimental ATP hydrolysis measurement, but may not have been present in the nucleation data presented because this was generated by a model-dependent computer simulation (Le Clainche et al. 2003).

We find that ATP hydrolysis and phosphate release from Arp2 (approximately 40 s) are more than an order of magnitude faster than debranching of Arp2/3-generated dendritic networks (approximately 1000 s) (Blanchoin et al. 2000b). The kinetics of phosphate release from Arp2 are also about an order of magnitude faster than phosphate release from actin (1/k_Pi release_ = 384 s for skeletal muscle actin; Melki et al. 1996), suggesting that, if phosphate release controls debranching, it is the phosphate release from the daughter actin filament that is important, not the phosphate release from Arp2. This is supported by the observation that phalloidin, which slows phosphate release from actin, slows filament debranching, and cofilin, which accelerates phosphate release from actin, accelerates filament debranching (Blanchoin et al. 2000b). Le Clainche et al. (2003) show that chromium-ATP Arp2/3 debranches more slowly than magnesium-ATP Arp2/3 and claim (but do not demonstrate) that chromium-ATP Arp2/3 releases phosphate more slowly. If chromium does slow the phosphate release from Arp2/3, in light of our data, this suggests that phosphate release from Arp2 may be a prerequisite for filament debranching—but is not a direct cause, since it occurs much too rapidly.

We previously showed that the Arp2/3 complex requires hydrolyzable ATP for nucleation activity (Dayel et al. 2001), and the current study adds weight to the hypothesis that ATP hydrolysis has a direct role in nucleation by showing that ATP is hydrolyzed by Arp2 upon nucleation. The separation of the Arps in the crystal structure and the very low nucleation rate of the unactivated complex probably reflect the tendency of Arp2 and Arp3 to remain separated in the absence of all the required nucleation promoting factors. This suggests that there is a large free energy barrier to the formation of an Arp2–Arp3 heterodimer. Our data indicate that there are two ways to overcome this energy barrier, both using the binding energy of actin: one using the combined binding energy of the two actin monomers at the pointed end of an actin filament during pointed-end capping, and the other the combined binding energy of the side of the mother filament, the VCA domain, and a single actin monomer. The surface area of the filament pointed end that would be buried by interaction with an Arp2–Arp3 dimer would be large (approximately 6800 Å^2^). This is consistent with the fact that in vitro the binding energy of this interface is sufficient to drive the interaction and promote the active conformation of the complex directly, even in the absence of VCA or a mother filament (Mullins et al. 1998). The binding of monomeric actin alone is insufficient to overcome the free-energy barrier, which ensures that the inactive conformation of the Arp2/3 complex is robust despite high cellular concentrations of actin. Because of the free energy of all the binding partners involved in nucleation, however, the energy of ATP hydrolysis may not be needed to stabilize the nucleus. Regardless, it is very likely that ATP hydrolysis on Arp2, like actin, provides a timing signal to the system. ATP hydrolysis on Arp2/3 would promote release of VCA from the complex and allow a new actin branch to move away from the site of its creation (Dayel et al. 2001). ATP hydrolysis may also regulate the timing of the interaction of the Arp2/3 complex with other binding partners such as cortactin and cofilin. Temporal regulation of these interactions is likely to be essential to construction of functional motile structures.

The Arp2/3 ATP hydrolysis assay presented here provides a novel assay for activation of the Arp2/3 complex that does not rely, as all previous assays have done, solely on actin polymerization. Pyrene–actin polymerization is only useful over a limited range of actin concentrations because at high concentrations, spontaneous assembly obscures Arp2/3-mediated nucleation. The pyrene–actin assay also has temporal limits since it rapidly uses up one of the factors necessary for Arp2/3 activation–monomeric actin. Our observation that ATP is hydrolyzed by Arp2 rapidly during, or soon after, the nucleation reaction means that we can use ATP hydrolysis on Arp2 as an assay to study the factors required to promote activation of the Arp2/3 complex. The fact that nonpolymerizable actin monomers are competent to stimulate hydrolysis enables us to investigate the conditions for Arp2/3 complex activation under a wider range of conditions. This system will be useful for further studies of the biophysics of Arp2/3-mediated actin assembly.

## Materials and Methods

### 

#### Purification of proteins

We purified Arp2/3 from Acanthamoeba castellini by a combination of conventional and affinity chromatography (Dayel et al. 2001). We flash-froze Arp2/3 complex in aliquots of approximately 40 μM in 10% glycerol, 0.5 μM TCEP, and 2 mM Tris (pH 8.0), and stored them at –80°C for later use. We purified actin from *Acanthamoeba* by the method of MacLean-Fletcher and Pollard (1980). Actin was stored in fresh G-buffer (0.5 μM TCEP, 0.1 μM CaCl_2_, 0.2 μM ATP, 2 mM Tris [pH 8.0]) and gel-filtered before use. Rat N-WASP VCA (398–502) and Human Scar1-VCA (489–559) with N-terminal 6His tags and TEV cleavage sites were bacterially expressed and purified by nickel affinity chromatography.

We prepared phalloidin-stabilized actin filaments by adding 1/10 volume of 10× KMEI to monomeric actin at room temperature for 20 min to initiate polymerization, then added twice the concentration of phalloidin and incubated for a further hour at room temperature (1× KMEI buffer: 50 mM KCl, 1 mM MgCl_2_, 1 mM EGTA, 10 mM Imidazole [pH 7.0]). We took care not to unintentionally shear the phalloidin-stabilized actin filaments by using wide-bore pipette tips.

#### Arp2/3 ATPase assay

We diluted freshly thawed aliquots of Arp2/3 to 2.0 μM in 1 mM MgCl_2_, 50 mM KCl, 10 mM Imidazole (pH 7.0) and added 6 μM γ-^32^P-labeled 8-AzidoATP (Affinity Labeling Technologies, Lexington, Kentucky, United States). After a 2-min incubation to allow nucleotide exchange, we crosslinked for 9 s using a UV hand lamp (312 nm; Fisher Scientific, Hampton, New Hampshire, United States), added 1 mM ATP and 1 mM DTT to quench the reaction and buffer exchanged into 1× KMEI plus 100 μM ATP, 1 mM DTT using a NAP5 column (Amersham Pharmacia Biotech, Little Chalfont, United Kingdom). We used the Arp2/3 for assays within 10 min of crosslinking. The same actin (including 7% pyrene–actin) was used for both ATP hydrolysis assays and correlative pyrene–fluorescence polymerization assays. We took ATPase time points by mixing 400 μl of the reaction mixture with premixed 400 μl of methanol and 100 μl of chloroform. We ran the precipitated protein on SDS-PAGE gel to separate the subunits and quantified ^32^P-labeling using a phosphoimager (Storm 840; Molecular Dynamics, Sunnyvale, California, United States). For phosphate cleavage assays, we quenched timepoints into 1/10 volume 26 M formic acid, spotted on cellulose TLC plates, and separated the components in 0.4 M KH_2_PO_4_ (pH 3.4). We separately ran ^32^P-ATP and ^32^P-ATP treated with apyrase as standards to confirm the separation of ^32^P-ATP and cleaved ^32^P, respectively (unpublished data). As an alternative method of quantifying cleaved ^32^P, phosphomolybdate was extracted as in Shacter (1984) and quantified using a scintillation counter. To distinguish the ADP-Pi state of Arp2 from the ADP state, the kinetics of phosphate release were measured by performing the reaction in the presence of 2 mM maltose and 2 U/ml maltose phosphorylase (Sigma-Aldrich, St. Louis, Missouri, United States), which uses only the released Pi to form glucose phosphate. Glucose phosphate was separated from free ATP, protein-ATP, and Pi using TLC.

#### Actin polymerization assays

We doped *Acanthamoeba* actin with 7% pyrene–actin to monitor actin polymerization by fluorescence (λ_ex_ = 365 nm, λ_em_ = 407 nm, 25°C) (Mullins and Machesky 2000). We calculated the number of ends produced over time from [ENDS] = (d[F-actin]/dt)/([free G-actin]*10 μM s^–1^) (cf. Zalevsky et al. 2001). Polymerization reactions were performed in G-buffer plus 1/10 volume 10× KMEI. The Ca^2+^ cation on monomeric actin was preexchanged with Mg^2+^ 30 s before use.

#### Microscopy

We prepared filamentous actin as above and stabilized filaments with stoichiometric Alexa-488 phalloidin (Molecular Probes, Eugene, Oregon, United States). We mixed 2 μM Alexa-488 phalloidin–F-actin with 20 nM Arp2/3, passed twice through a 30-gauge needle to shear the filaments, and incubated at room temperature. Timepoints were taken by diluting 500-fold and rapidly applying to poly-L-lysine–coated coverslips for visualization. Filament images were quantified for length distribution and branch frequency by a custom MATLAB (MathWorks Inc., Natick, Massachusetts, United States) routine.

## Abbreviations

Arp: Actin-related protein
ATP: adenosine triphosphate
N-WASP: neuronal Wiskott–Aldrich syndrome protein
Pi: inorganic phosphate
SDS-PAGE: sodium dodecyl sulphate polyacrylamide gel electrophoresis
TLC: thin-layer chromatography
VCA: verprolin-homology
WASP: Wiskott–Aldrich syndrome protein
WH2: WASP homology 2
